# Does Motivation in Physical Education Have an Impact on Out-of-School Physical Activity over Time? A Longitudinal Approach

**DOI:** 10.3390/ijerph17197258

**Published:** 2020-10-04

**Authors:** Djenna Hutmacher, Melanie Eckelt, Andreas Bund, Georges Steffgen

**Affiliations:** 1 Department of Behavioural and Cognitive Sciences, University of Luxembourg, Campus Belval, 11, Porte des Sciences, Esch-sur-Alzette, L-4366 Luxembourg, Luxembourg; georges.steffgen@uni.lu; 2 Department of Education and Social Work, University of Luxembourg, Campus Belval, 11, Porte des Sciences, Esch-sur-Alzette, L-4366 Luxembourg, Luxembourg; melanie.eckelt@uni.lu (M.E.); andreas.bund@uni.lu (A.B.)

**Keywords:** self-determination theory, physical activity, physical education, trans-contextual education, longitudinal design

## Abstract

Previous research based on the trans-contextual model proposes that autonomous motivation in physical education (PE) is transferable to an out-of-school leisure-time (LT) context. However, only cross-sectional and unidirectional analyses have been conducted. The present study used a longitudinal design assessing *N* = 1681 students (*M* = 14.68 years) on two occasions, measuring the following constructs: perceived need for support in PE, motivational regulation during PE and LT, attitude, subjective norm, perceived behavioral control, intention, and physical activity behavior. Findings based on mixed effect models revealed that autonomy, competence, and relatedness support of the PE teacher were positively related to autonomous motivation. Moreover, similar motivational regulation types were found to significantly cross-lag across contexts. Through longitudinal mediation analyses, further support for the impact of autonomous motivation on physical activity, mediated by intention, attitude, and perceived behavioral control, was found. Suggestions for educational stakeholders regarding how to promote students’ autonomous motivation are provided.

## 1. Introduction

Physical education (PE) holds a unique and advantageous position in being able to address a significant number of children and adolescents. This, in turn, highlights its importance to promote competencies in school that will be beneficial to and implemented by the students also in their everyday life. Generally, one of the main components to ensure learning, or the intention to act, is one’s inner motivation to do so [1,2]. In their multi-theoretical trans-contextual model, Hagger, Chatzisarantis, Culverhouse, and Biddle [3] postulate that the promotion of autonomous motivation in classrooms persists towards similar activities in contexts outside the school. Thus, students’ autonomous motivation towards activities generated during PE may be transferred towards similar activities outside the classroom, such as physical activities in their leisure-time (LT). This trans-contextual transfer is highly important, especially when considering the overall continuing decrease in physical activity among youth [4].

More specifically, in their trans-contextual model, the authors propose three assumptions: (1) if the students perceive their PE teacher as need-supportive, this will positively influence their autonomous motivation, (2) autonomous motivation in PE will positively influence the students’ autonomous motivation in an out-of-school context, and (3) autonomous motivation in an out-of-school context predicts future intentions to engage in out-of-school physical activities. In general, results of previous studies support these assumptions [5,6]. However, these studies tested the trans-contextual model only by means of correlative analyses, without considering the predicted causal directions. Therefore, the overarching aim of this study is to test these assumptions in a longitudinal design. In the following sections, the three assumptions of the trans-contextual model will be discussed in detail, as well as the resulting research questions for the present study.

### 1.1. Assumption 1: The Students’ Perceived Basic Needs Support by the Teacher Influences the Students’ Motivation in PE

The self-determination theory (SDT), a macro theory of human motivation [7], does not treat motivation as a unitary concept but differentiates between several types of motivation on a continuum, with the most central distinction being made between intrinsic and extrinsic motivation. Unlike different perspectives seeing extrinsic motivation as invariantly non-autonomous, SDT proposes that extrinsic motivation can greatly vary in its level of autonomy [8]. In this sense, SDT defines three types of autonomous motivation. Firstly, *intrinsic motivation* refers to the most autonomous form of the continuum and reflects the engagement in an activity for the inherent satisfaction of the activity itself, because doing so leads to experience ownership over one’s actions and consistency between one’s behavior and authentic self. Intrinsic motivation is the only behavioral regulation type, which is performed in the absence of any external contingency. Secondly, another type of autonomous motivation is *identified regulation*. Identification reflects a conscious valuing of a behavioral goal or regulation, such that the individual is engaging in activities because they serve self-endorsed outcomes and are personally important. The third type of autonomous regulation, which reflects the most autonomous form of extrinsic motivation, is *integrated regulation*. Integration occurs when identified regulations are fully assimilated to the self, which means that integrated regulated actions are perceived to be entirely consistent with self-endorsed values and needs. Actions characterized by integrated motivation share many qualities with intrinsic motivation, although they are still considered extrinsic because they are performed to attain separable outcomes rather than for their inherent enjoyment [8]. In contrast to autonomous motivation, two controlled types of motivation are proposed by SDT, which reflect behavioral engagement for reasons of more externally regulated motivators, such as obligation or reinforcement. The first extrinsically motivated and least autonomously motivated behavior is *external regulation*, which reflects behaviors that are performed to satisfy an external demand in order to receive a reward or avoid punishment. The reason to engage in the behavior is perceived as controlled and inconsistent with the individual’s authentic sense of self. Lastly, *introjected regulation* involves taking in a regulation to the self but not fully accepting it as one’s own. This is a relatively controlled form of regulation, in which behaviors are performed to avoid guilt or to promote contingent self-worth. Even though introjected regulated actions are driven by the individual itself, they represent experiences as other-endorsed rather than as a part of the self. Both intrinsic and extrinsic motivation presuppose that an individual is motivated in the first place. In this sense, and in contrast to these motivation types, SDT defines *amotivation* as a state of lacking any intention to act.

The theoretical assumptions of SDT have been applied to a wide range of life domains. A more autonomously regulated form of motivation is considered to be associated with the persistence of self-determined learning and behavioral activities, and was found to be related to various positive outcomes. These outcomes range from academic attainment, performance, knowledge, psychological wellbeing, and happiness to the intention to become physically active [9,10,11,12], and, thus, represent a particularly important factor in education. 

As a substantial basis for energizing and directing action from a controlled towards an autonomous motivation, Deci and Ryan [13] use the concept of three innate psychological needs for autonomy (sense of control of own behavior), relatedness (feeling connected with significant others), and competence (feeling of being able to carry out a behavior). With regard to PE, previous studies suggest that teachers, who act in a need-supportive manner, facilitate students’ need satisfaction and, thus, generate motivation that is more autonomous and, hence, internally motivated [5,14,15]. 

Interestingly, previous research has primarily focused on the promotion of the support of autonomy [16]. As the satisfaction of all three needs in SDT [13] is described as innate and fundamental for intrinsic motivation, we assume that, consequently, the support of all three different basic needs will positively affect the students’ autonomous motivation. This is why, in the present study, the students’ perceived support of all three basic needs by the PE teacher will be analyzed over time in order to study their distinct influence on intrinsic motivation and identified regulation. For instance, providing choice for the students in class (autonomy support), encouraging students to set realistic goals (competence support), and creating a climate where students are encouraged to interact with the teacher and classmates (relatedness support) should promote self-determined motivation in class. Furthermore, as contextual motivation is a dynamic process, changes over time are very likely to occur [17]. By employing a mixed effects model approach, we are able to analyze continuous responses in our longitudinal design in order to analyze the proposed relations via a robust method controlling for fixed and random effects [18].

### 1.2. Assumption 2: School-Related PE Motivation Persists in an Out-of-School (LT) Context

The second main goal of this study is to uncover larger persisting effects of motivational regulation types beyond the school context (i.e., towards an out-of-school context, e.g., physical activity during LT) [6]. This transfer of the motivational regulation from one context towards another has been discussed in two theoretical approaches. First, continuing motivation [19] is defined as the tendency to return to and continue working on tasks away from the instructional context. For instance, children may learn the relevant techniques of playing a particular sport in class, but becoming a good player requires them to continue practicing without instructions over and above that, namely, also in an out-of-school context [19]. Second, the hierarchical model of intrinsic and extrinsic motivation [20] postulates that different motivational types should be inter-correlated or, in other words, persist over contexts. Hagger and colleagues [3] describe this inter-correlation between contexts in their trans-contextual model, using the transfer of motivation from a school context towards an out-of-school context as showcase. In their meta-analytic path analysis, Hagger and Chatzisarantis [6] found a significant and large positive relation between autonomous motivation during PE and autonomous motivation of physical activity during LT. This finding is important insofar as a successful promotion of autonomous motivation in schools might persist in an out of-school context, which is crucial for continuing self-determined regulation of behavior [21]. 

As earlier research focused on cross-sectional path analyses to test the assumptions of the trans-contextual model, in this study, we want to analyze the often criticized proposed unidirectional paths in a longitudinal setting [22]. Based on Vallerands’ hierarchical model [20], we assume that, beyond the assumptions of Hagger and colleagues [3], not only similar motivation types in PE will be associated with LT over time, but that also similar motivation types in LT will influence similar motivation types in PE. In other words, we suspect that the promotion of autonomous motivation in class affects autonomous motivation in out-of-school physical activities, and vice versa. Although controlled types of motivation and amotivation are significant factors present in PE and LT, as, for example, repetitive and boring exercises might very probably be experienced in both school and LT, we assume that also controlled types of motivation and amotivation will be intercorrelated over time and context. Thus, in the present study, we hypothesize that all five types of motivational regulation measured in this study (i.e., amotivation, external regulation, introjected regulation, identified regulation, and intrinsic motivation) positively influence their counterparts across PE and LT, and might exert a negative influence on motivation types from the other end of the continuum (e.g., intrinsic motivation in LT might be negatively related to external regulation in PE over time). In order to analyze these proposed relationships, a cross-lagged panel model is employed [22].

### 1.3. Assumption 3: Motivation in LT Influences Physical Activity Behavior in LT

Finally, as the promotion of autonomous motivation in PE should persist in LT, the question whether autonomous motivation in LT has an impact on actual physical activity behavior in LT remains unanswered. However, answering this question becomes crucial when considering that only one third of adolescents from 146 countries reach the World Health Organization (WHO) guidelines of being physically active for at least 60 minutes a day [4]. In their trans-contextual model, Hagger and colleagues [3] refer to the theory of planned behavior (TPB) [23] in order to analyze and understand volitional and intentional behavior. According to the TPB, an individual’s intention towards a specific behavior is the strongest predictor of behavior. Intention is proposed to be mediated by the attitudes towards the specific behavior, the subjective norms (pressure placed by significant others towards the behavior), and the perceived behavioral control (one’s own capability to do it). In turn, perceived behavioral control is envisaged to have a direct effect on behavior. Even though this social-cognitive model successfully explains a substantial proportion of variance in physical activity intentions and behavior in studies with adolescents [24], the model does not consider global, goal-related motives, which may act as sources of information in the formation of intentions [25]. In order to expand the question of “what”, as addressed in social-cognitive frameworks, the SDT was found to be adaptable in implementing the question of “why” individuals form intentions, attitudes, subjective norms, and perceptions of their behavioral control. Recent studies provided support for the influence of the different motivational regulation types (generalized motives) towards the situation-specific decision-making variables and found substantial evidence to combine the two theoretical models [25]. 

The third aim of the present study is to extend the previous findings of autonomous and controlled motivation types towards the TPB in a longitudinal mediation analysis [26]. As internal motives have been found to act as an autonomous perceived locus of control in the formation of attitudes and perceived behavioral control [27], we anticipate that intrinsic motivation and identified regulation will significantly and positively influence attitudes, perceived behavioral control, and intentions over time. In addition, physical activity behavior is suspected to be mediated by intentions, attitudes, and perceived behavioral control. Furthermore, it is expected that external regulation and introjected regulation will exert a significantly negative influence on attitudes and perceived behavioral control, as these regulation types consider controlling motives, in the sense that pressure to comply may hinder one’s willingness to perform an action [25]. Finally, external and introjected regulation are hypothesized to exert a significant and positive influence on subjective norms, as subjective norms are generally constructed as representative entities of social pressures to engage in behavior [3].

Taken together, we assume, that, firstly, (Assumption 1) the students’ perceptions of all three psychological basic needs will positively influence autonomous motivation (i.e., identified regulation and intrinsic motivation) in PE over time. Secondly, (Assumption 2) we assume that autonomous, controlled motivation (i.e., external and introjected regulation) and amotivation in PE will both positively influence their respective counterparts in LT, and vice versa. Furthermore, autonomous motivation in PE is assumed to influence controlled motivation in LT negatively, and vice versa, while controlled motivation in PE is assumed to influence autonomous motivation in LT negatively, and vice versa. Finally, (Assumption 3) we assume that autonomous motivation will influence the intention and physical activity behavior over time, which we believe to be mediated by attitudes and perceived behavioral control, while controlled motivation should affect subjective norms over time.

## 2. Methods

### 2.1. Participants

In total, *N* = 1877 students aged 10 to 23 years (*M* = 14.74 years old; 922 females (49.1%)) were tested at the first wave of the data collection (T1). Subsequently, *N* = 194 (10.4%) students dropped out at T2 (e.g., due to illness) and were not retained in the analyses. As attrition rates from 30 to 70% are often reported in longitudinal studies [28], a dropout rate of around ten percent can be considered negligible. Nevertheless, the *N* = 194 students reported significantly (*p* < 0.05) lower rates of intrinsic motivation and identified regulation in PE and LT, subjective norms, autonomy, relatedness, and competence support of the PE teacher, while they rated their external regulation (PE) and amotivation (PE and LT) significantly higher. No remaining scales differed (all *p*-values > 0.05). Of the remaining *N* = 1681 students (853 females (50.7%); age of *M* = 14.68 (*SD* = 2.66) years) included in this study, *N* = 1140 (67.8%) students were born in Luxembourg, *N* = 224 (13.3%) in Portugal, and *N* = 317 (18.9%) in other countries. Additionally, *N* = 366 students (21.8%) went to elementary schools, while *N* = 1315 (78.2%) students (376 (22.4%) from the 7th, 459 (27.3%) from the 9th, and 480 (28.6%) from the 11th grade) went to secondary schools. The nine elementary and five secondary schools were selected with the purpose of including each geographical region of the country of Luxembourg. Overall, *N* = 1060 (63.1%) students chose to fill out the questionnaire in German, *N* = 555 (33.0%) in French, and *N* = 66 students (3.9%) switched between the languages during the survey.

### 2.2. Measures

All questionnaires used in this study were translated from English into German and French using the back-translation technique [29]. Two psychologists from the field of sport psychology separately translated all the scales from English to German and French, while two different psychologists translated the scales back to English. Lastly, the scales were adapted and revised in wording and syntax by a French and German teacher.

*Need support*. In order to assess the participants’ perceived autonomy, competence, and relatedness support of the PE teacher, 16 items out of the 24 items were used, as described in the study of Standage, Duda, and Ntoumanis [30]. All items were preceded by the stem “In this PE class…” and responses were provided on a 7-point scale (1 = not agree at all, and 7 = totally agree). In order to achieve equivalence in the number of items assessing each subscale, seven out of 15 items were used for autonomy support (*α* = 0.82). These items were chosen independently and agreed upon by two experienced sport psychologists familiar with the SDT and based on their content-related proximity to the well-established questionnaire Perceived Autonomy Support Scale for Exercise Settings (PASSES) [31]. The following items were included: “we feel that the PE teacher provides us with choices and options”; “the PE teacher shows confidence in our abilities to do well in PE”; “the PE teacher makes sure we really understand the goals of the lesson and what we need to do”; “the PE teacher encourages us to ask questions”; “the PE teacher answers our questions fully and carefully”; “the PE teacher tries to understand how we see things before suggesting new ways to do things”; “we feel able to share our feelings with the PE teacher”. Four items were used for competence support (*α* = 0.83; example item “the PE teacher makes us feel like we are able to do the activities in class”) and five items for relatedness support (*α* = 0.81; example item “we feel that the PE teacher encourages us to work together in class activities”). Confirmatory factor analyses revealed a good model fit for the three factors, *χ*^2^ = 1141.94; *df* = 101; *p* < 0.001; RMSEA (root mean squared error of approximation) = 0.078; 90% CI (confidence interval) = [0.074; 0.082]; SRMR (standardized root mean square residual) = 0.04; CFI (comparative fit index) = 0.92; TLI (Tucker–Lewis index) = 0.91. Factor loadings ranged from 0.45 to 0.73 for autonomy support, from 0.63 to 0.74 for relatedness support, and from 0.71 to 0.77 for competence support.

*Motivation in PE*. The revised Perceived Locus of Causality Scale (PLOC-R) [32] was used to measure the behavioral regulation in PE. Following the stem “I participate in PE…”, the students provided their answers on 20 items with a 7-point scale (1 = not agree at all, and 7 = totally agree). Each of the subscales consisted of four items for amotivation (*α =* 0.80; example item “But I don’t see why I should have PE”), external regulation (*α =* 0.71; example item “Because I’ll get into trouble if I don’t”), introjected regulation (*α* = 0.68; example item “Because I would feel bad about myself if I didn’t”), identified regulation (*α* = 0.85; example item “Because it is important to me to try in PE”), and intrinsic motivation (*α* = 0.82; example item “Because PE is fun”). Factor loadings ranged from 0.68 to 0.74 for amotivation, from 0.48 to 0.73 for external regulation, from 0.58 to 0.69 for introjected regulation, from 0.74 to 0.80 for identified regulation, and from 0.66 to 0.80 for intrinsic motivation. For further psychometric details, please refer to Hutmacher, Eckelt, Bund, and Steffgen [33].

*Motivation in LT*. The 19-item Behavioral Regulation in Exercise Questionnaire (BREQ-II) [34] was used to assess the behavioral regulation in LT. Students provided their answers on a 7-point scale (1 = not agree at all, and 7 = totally agree). The subscales amotivation (*α* = 0.86; example item “I think exercising is a waste of time”), external regulation (*α* = 0.80; example item “I exercise because other people important to me say I should”), identified regulation (*α* = 0.76; example item “I value the benefits of exercise”), and intrinsic motivation (*α* = 0.87; example item “I exercise because it’s fun”) each consisted of four items, while the subscale introjected regulation (*α* = 0.69; example item “I feel guilty when I don’t exercise”) contained three items. Factor loadings ranged from 0.73 to 0.84 for amotivation, from 0.66 to 0.77 for external regulation, from 0.59 to 0.72 for introjected regulation, from 0.47 to 0.79 for identified regulation, and from 0.72 to 0.85 for intrinsic motivation (please refer to Hutmacher, Eckelt, Bund, and Steffgen [33] for further psychometric details).

*Constructs of the theory of planned behavior (TPB)*. Items from Hagger and colleagues [3], which were adapted according to the procedure proposed by Ajzen and Madden [35], were used. The *attitude* to engage in physical activity (*α* = 0.90) was recorded using seven bipolar adjectives (e.g., unpleasant–pleasant) with the introductory stem “I find being physically active in my free time at least 60 minutes a day ...”. Factor loadings ranged from 0.66 to 0.80. All adjectives were rated on a 7-point semantic differential scale, with higher values representing the positive adjective. *The perceived behavioral control* to become physically active regularly in the free time was recorded using three items (*α* = 0.77). Factor loadings ranged from 0.70 to 0.75. Two items (example item “I have full control over whether I am active in my free time for at least 60 minutes a day”) were rated on a 7-point scale (1 = not agree at all, and 7 = totally agree), and the item “How much control do you have about being physically active in your free time for at least 60 minutes a day” was rated on a 7-point scale from 1 (“no control at all”) to 7 (“complete control”). To assess the *subjective norm*, three items based on the injunctive norm (e.g., “People who are important to me encourage me to be physically active in my free time”) were used. These items showed an internal consistency of *α* = 0.65 and were rated on a 7-point scale (1 = not agree at all, and 7 = totally agree). The *intention* to become physically active was measured using two items (*α* = 0.79). The item “I intend to be physically active for at least 60 minutes a day for the next 5 weeks” was rated on a 7-point Likert scale (1 = not agree at all, and 7 = totally agree), and the second item “I intend to be physically active at least 60 minutes a day with the following regularity” was recorded on an 8-point Likert scale (0 = never, 7 = daily). The *subjectively perceived physical activity* behavior during LT was assessed via two items, “On how many days of a regular week are you physically active for at least 60 minutes?” and “On how many days were you physically active for at least 60 minutes over the last seven days?”, on an 8-point scale (0 = never, to 7 = on each day).

### 2.3. Procedure

Each of the two data collection waves lasted for around two months and was performed digitally via self-report questionnaires using the secured platform OASYS [36] (University of Luxembourg, Esch-sur-Alzette, Luxembourg). Due to the advantage of digital testing, no missing data need to be reported. The questionnaires were filled out at school during class on a computer or tablet under the continuous supervision of trained research assistants. The first data collection (T1) took place during the first trimester of the participants’ school year in autumn. The second data collection (T2), consisting of the same questionnaires, was performed six months later during the third trimester of the participants’ school year in spring. Assessing the students twice during one school year leads to a reduced percentage of dropouts, while, simultaneously, six months can be considered as a sufficient period to capture potential substantial change in adolescents’ values and behaviors (e.g., physical maturation) [37]. Ethical approval was provided by the Ethics Review Panel of the University of Luxembourg. Signed informed consent forms were required from all participants in order to take part in our study, while written permission was additionally needed from the legal representatives of all participants younger than 16 years.

### 2.4. Statistical Analysis

As a longitudinal design was chosen, measurement invariance (MI) has to be controlled for, since the different scales should measure the same construct over time [38]. Given the sensitivity of the χ^2^ statistics to sample size, model fit assessment was primarily based on the remaining fit indices. According to the cut-off value criterion of Cheung and Rensvold [39] suggesting a ΔCFI (change in the Comparative Fit Index) of −0.01, we found evidence that all reported scales showed strict measurement invariance (equal pattern loadings, loading values, intercepts of each item, and residual variances) over the two measurement points. Furthermore, the univariate and multivariate distributions of the items and the scales’ internal reliability and factorial structure were tested and revealed good psychometric properties. As MI over time is given, the psychometric properties are reported only for T1. A minimum value of 0.40 was accepted for factor loadings [40].

In order to analyze Assumption 1, a generalized linear mixed model (GLMM) [18] was conducted to model within-subject correlations over time, while simultaneously controlling for age and sex. The specified model can be defined as$\;Y_{ij}=\beta_{o}+\;b_{0a}+b_{0r}+b_{0c}+\;\beta_{1T}+\;\beta_{2A\;}+\;\beta_{3S\;}$, while$\;Y_{ij}$ denotes the logit of intrinsic motivation or identified regulation, $\beta_{1T},$ $\beta_{2A}\;$and $\beta_{3S}\;$reflect the three respective fixed terms time, age, and sex, and $\beta_{o}$ represents the *general intercept*. The three predictors, with varying intercepts on $Y_{ij}$ being controlled for by the GLMM approach, are listed as $b_{0a}\;$for autonomy support, $b_{0r}\;$for relatedness support, and as $b_{0c}\;$for competence support. Secondly, to analyze the interactions and reciprocal influences between the variables over a two-time period, a Cross-Lagged Panel Model (CLPM) was used, as described by Mund and Nestler [22], for Assumption 2. Thirdly, a longitudinal mediation model, as described in Jose [26], was conducted addressing Assumption 3. Responses to items measuring the same construct were averaged for use in the correlation and path analyses. Exogenous variables were allowed to correlate, as well as the disturbances of the endogenous variables. Model fit of the CLPM, the confirmatory factor analysis (CFAs), and the longitudinal mediation model was evaluated with the root mean squared error of approximation (RMSEA), accompanied by a 90% confidence interval (CI), the standardized root mean square residual (SRMR), comparative fit index (CFI), and Tucker–Lewis index (TLI). Based on the cut-off values from Hu and Bentler [41], a reasonable fit was accepted (RMSEA < 0.08; SRMR < 0.12; CFI > 0.90; TLI > 0.90).

In order to perform the statistical analyses, the 25th version of the “Statistical Package for the Social Sciences” (SPSS) [42] (IBM Corp, Armonk, NY, USA) software was used for data transformation, descriptive analyses, and independent sample t-tests. Version 3.6.1 of R [43] (R Foundation for Statistical Computing, Vienna, Austria) and the package lme4 [44] were used for the GLMM analysis (Assumption 1), and the package lavaan [45] was used for the CFAs. In addition, Amos 26 [46] (IBM SPSS, Chicago, IL, USA) was used to perform the CLPM and longitudinal mediation model (Assumptions 2 and 3). Finally, measurement invariance analyses were performed with Mplus 5.0 [47] (Muthén & Muthén, Los Angeles, CA, USA).

## 3. Results

### 3.1. Preliminary Analysis

As the measures for motivational regulation in PE and LT share equal constructs, one might have doubts with regard to the construct validity of these two very similar scales. In order to address this question, two CFAs were conducted and tested against each other. The first congeneric model consisted of five factors, on which all indicators of the perceived locus of causality in PE and LT were loaded. For example, the items of intrinsic motivation from PE and LT were loaded on one overarching factor. This congeneric model with five factors revealed an unsatisfactory model fit, *χ*^2^ = 6931.12.; *df* = 655; *p* < 0.001; RMSEA = 0.075; 90% CI = [0.074; 0.077]; SRMR = 0.098; CFI = 0.741; TLI = 0.722, with factor loadings ranging between 0.31 and 0.79. In a second model, the indicators of the perceived locus of causality in PE and LT were loaded on ten distinct factors and revealed a satisfactory model fit, *χ*^2^ = 2281.90.; *df* = 620; *p* < 0.001; RMSEA = 0.040; 90% CI = [0.038; 0.041]; SRMR = 0.054; CFI = 0.931; TLI = 0.922, with factor loadings ranging between 0.46 and 0.85. When comparing these models, the model with ten factors fitted significantly better (Δ*χ*^2^ = 3351.5, *p* < 0.001).

The correlation coefficients and descriptive statistics among all psychological measures are presented in Table 1. Similar scales correlated higher beyond the two different questionnaires, which confirms convergent validity. For instance, intrinsic motivation in PE (PLOC-R) correlated with intrinsic motivation in LT (BREQ-II). In addition, simplex pattern correlations between the five factors of the continuum for motivational regulation in PE and LT were found and, thus, support the theoretically expected relationship pattern between the subscales [32,34]. For example, a greater correlation was revealed between intrinsic motivation during PE and identified regulation in PE (*r* = 0.73) than with introjected regulation in PE (*r* = 0.12). All correlations according to the TPB were obtained in the expected way [23]. As such, attitudes, subjective norms, and perceived behavioral control towards physical activity were positively related to the intention of getting physically active, which, in turn, was related to actual physical activity.

Participants subjectively reported on average *M* = 3.21 (*SD* = 1.97) days of at least 60 minutes of physical activity at T1 and *M* = 3.32 (*SD* = 1.99) at T2. Overall, 8.3% of the adolescents at T1 and 9.5% at T2 reported being physically active for at least 60 minutes every day.

### 3.2. Assumption 1: The Students’ Perceived Basic Needs Support by the Teacher Influences the Students’ Motivation in PE

In order to test if the support of the three basic psychological needs had an impact on intrinsic motivation and identified regulation, two GLMM models were conducted controlling for time, age, and sex. Overall, results of the GLMM analyses, which are presented in Table 2, showed that the students’ perceived autonomy support, relatedness support, and competence support of the PE teacher significantly predicted intrinsic motivation and identified regulation. Competence support was the strongest predictor for both intrinsic motivation (*β* = 0.27; *p* < 0.001) and for identified regulation (*β* = 0.35; *p* < 0.001). The three predictors explained 26% of the variance in intrinsic motivation and 27% of variance in identified regulation, while the full GLMM was able to explain 67% of the variance in intrinsic motivation and 61% in identified regulation when also taking time, age, and sex into account.

### 3.3. Assumption 2: School-Related PE Motivation Persists in an Out-of-School (LT) Context

**Figure 1:**
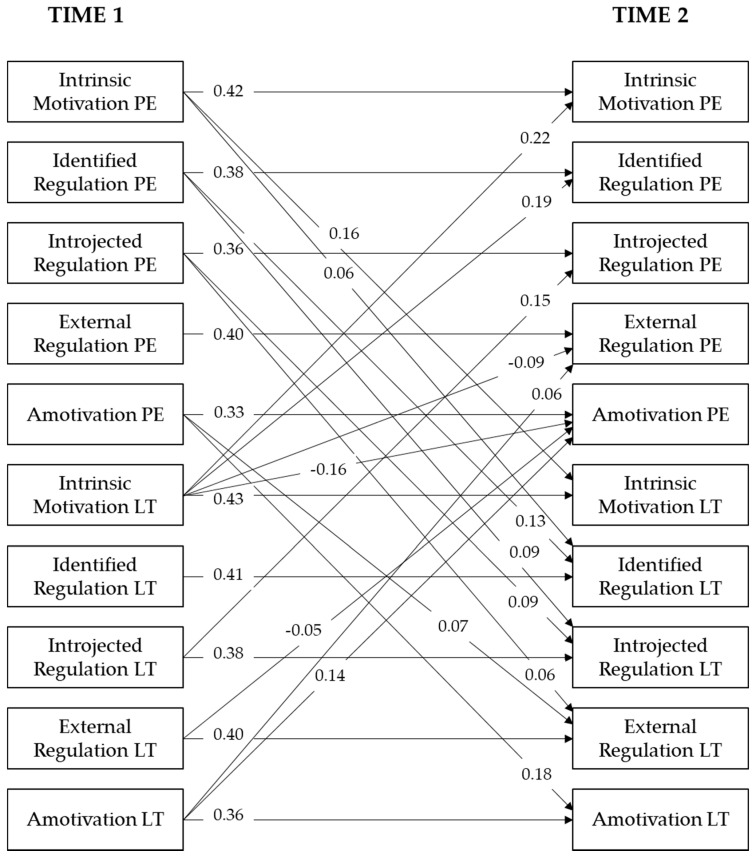
Standardized paths of a cross-lagged panel model of the five different factors of motivation in physical education (PE) and in leisure-time (LT). All cross-lagged paths between the five motivational types in PE and LT were calculated. Only significant paths are presented (*p* < 0.05).

In order to analyze the transfer of motivation from the context of PE towards the context of LT, as well as from LT to PE, the impact of the students’ perceived intrinsic motivation, identified regulation, introjected regulation, external regulation, and amotivation towards PE was tested against the same factors towards LT, and vice versa, in a cross-lagged analysis. The respective significant paths are shown in Figure 1.

Except for external regulation in PE, external regulation in LT, and identified regulation in LT, all directly related motivational types influenced each other over time and context. The model showed a good fit to the data, *χ*^2^ = 450.71; *df* = 40; *p* < 0.001; RMSEA = 0.078; 90% CI = [0.072; 0.085]; SRMR = 0.05; CFI = 0.98; TLI = 0.90. Overall, the model explained a considerable proportion of variance in all six outcomes at T2, for intrinsic motivation in PE (*R*^2^ = 0.30), identified regulation in PE (*R*^2^ = 0.27), introjected regulation in PE (*R*^2^ = 0.21), external regulation in PE (*R*^2^ = 0.22), amotivation in PE (*R*^2^ = 0.24), intrinsic motivation in LT (*R*^2^ = 0.32), identified regulation in LT (*R*^2^ = 0.29), introjected regulation in LT (*R*^2^ = 0.23), external regulation in LT (*R*^2^ = 0.22), and amotivation in LT (*R*^2^ = 0.21).

### 3.4. Assumption 3: Motivation in LT Influences Physical Activity Behavior in LT

**Figure 2:**
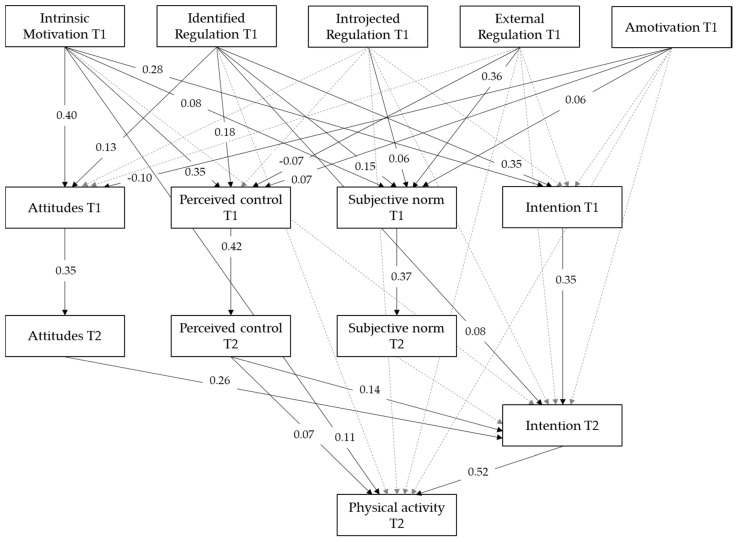
Longitudinal mediation model of the five different factors of motivation in leisure-time (LT) on intention and physical activity mediated by attitude, perceived behavioral control, and subjective norm. All calculated paths are presented: solid arrows represent significant paths (*p* < 0.05), while dashed arrows represent non-significant paths (*p* > 0.05).

In order to analyze the impact of autonomous motivation, controlled motivation, and amotivation on intentions and physical activity in LT, and the potential mediations of attitude, subjective norm, and perceived behavioral control, a longitudinal mediation analysis was conducted. The respective modeled paths are presented in Figure 2. Statistical significance was obtained for all paths in line with the proposed assumptions. As a direct significant path from intrinsic motivation towards physical activity behavior was present, the two serial mediation paths from intrinsic motivation on attitudes (perceived behavioral control) on intention and finally on behavior were calculated and revealed significance (*β_attitude_* = 0.02; *p* < 0.01; *β_control_* = 0.01; *p* < 0.05). Additionally, as identified regulation directly predicted the intention of becoming physically active, two further mediation paths from identified regulation on attitudes (perceived behavioral control) and on intention were conducted and showed significance (*β_attitude_* = 0.01; *p* < 0.001; *β_control_* = 0.01; *p* < 0.05). The further five full mediation analyses of identified regulation, external regulation, and amotivation on physical activity behavior were conducted and found to be significant (*p* < 0.05). The model showed a good fit to the data, *χ*^2^ = 323.60; *df* = 30; *p* < 0.001; RMSEA = 0.076; 90% CI = [0.069; 0.084]; SRMR = 0.06; CFI = 0.97; TLI = 0.91. Overall, the model explained a considerable proportion of variance in all nine outcomes, for attitude at T1 (*R*^2^ = 0.28), perceived behavioral control at T1 (*R*^2^ = 0.23), subjective norm at T1 (*R*^2^ = 0.24), attitude at T2 (*R*^2^ = 0.12), perceived behavioral control at T2 (*R*^2^ = 0.18), subjective norm at T2 (*R*^2^ = 0.14), intention at T1 (*R*^2^ = 0.37), intention at T2 (*R*^2^ = 0.40), and physical activity at T2 (*R*^2^ = 0.40).

## 4. Discussion

The overarching aim of this study was to investigate if motivational regulation during PE is related to the motivational regulation of physical activity during the students’ LT, and if their perceived need-support from the teacher enhances this transfer between the school and out-of-school context. Therefore, the assumptions of the trans-contextual model [6] were analyzed in a longitudinal design.

Based on the first assumption (1), we found that the students’ perceived autonomy, competence, and relatedness support from the PE teacher were, as expected, related to intrinsic motivation and identified regulation in PE over time. Thus, while controlling for time, age, and sex, all students perceived higher autonomous motivational rates when they perceived their PE teacher as need-supportive. Interestingly, in accordance with our theoretical expectations and in addition to previous research [16], we found that over and above the support of autonomy support during PE, competence support and relatedness support functioned as significant factors to impact autonomous motivation in PE. In line with the findings of Kiemer, Gröschner, Kunter, and Seidel [48], we found that, over time, competence support functioned as an important prerequisite for students’ self-determination in school. The authors further suggest that it is important to consider how the teacher supports competence, as communication in a context of respect, with a meaningful rationale, is crucial. In this sense, it is not surprising that, next to competence, our results revealed that also the support of relatedness, thus, a good social climate in class, significantly improved autonomous motivation. In sum, we therefore suggest that PE teachers support autonomy (i.e., providing choice of activities), competence (i.e., setting realistic goals and promoting a feeling of ability to succeed), and relatedness (i.e., spreading a feeling of respect and kindness) simultaneously, in order to significantly strengthen the self-induced (i.e., internal) motivational regulation of their students. Furthermore, we suggest implementing competence and relatedness support in the trans-contextual model, as we believe it will provide incremental value to the model, as 67% of intrinsic motivation and 61% of identified regulation were explained.

According to the second assumption (2), the postulated transfer of motivational regulation between contexts could be confirmed. Indeed, as proposed in the trans-contextual model, we found that autonomous motivation (i.e., intrinsic motivation and identified regulation) in a school context (PE) was directly related to autonomous motivation in LT [6] and vice versa. These results confirm that motivational regulation is transferable to similar activities in a different context [20]. Furthermore, these findings point out the importance of education, as teachers’ encouragement of their students’ autonomous motivation during PE is likely to persist in LT. Furthermore, intrinsic motivation in LT over time was related to intrinsic motivation in PE, meaning that students, who like to be physically active during LT because they like the activity itself, are also likely to implement this intrinsic motivation during PE. In addition, intrinsic motivation in LT was negatively related to external regulation and amotivation in PE, showing that students who appreciate physical activity in their free time do no not value external motivators in PE. Opposed to the transfer from PE to LT, identified regulation was not related from LT to PE over time. A possible explanation for the absence of this transfer might be that students identify with different values in LT compared to PE. Successful internalization, termed identification, involves the integration of formerly external regulations into one’s sense of self, typically in the form of important personal values, which are dynamic and dependent upon social-contextual support [49]. Likewise, it might be the case that, in LT, identified regulatory motives rather represent the benefits of physical activity in a more global setting, while in PE, the students were primarily asked whether it is valuable for them to try and accomplish new activities. These results suggest that the identified values seem to be transferable only from a more specific school-related context to more global physical activity values in LT, but, at the same time, these values from an out-of-school context were not implemented in PE.

Additionally, we found that introjected regulation and amotivation in PE were related to introjected regulation and amotivation in LT over time, which was also the case for the transfer from LT towards PE. A cross-contextual transfer of introjected regulation may imply that if students become physically active based on internalized external contingencies, they are very likely to transfer these regulatory motives between the PE and LT context. Furthermore, if students are not motivated towards physical activities at all, they tend to remain amotivated in both contexts. Interestingly, external regulation was not transferred over time and context. An explanation for this absence might be that external regulatory motives differ between the school and out-of-school context, as, for instance, external drivers in school are rules or grades, while, in LT, external drivers are significant others, who want one to be physically active. However, these external motives in LT negatively influenced amotivation in PE over time, meaning that the pressure of significant others to become physically active reduced the feeling of not knowing why physical activity is of importance (i.e., not a waste of time) in school.

Finally, addressing the third assumption (3), we found evidence for the claims made by the trans-contextual model, namely that intrinsic motivation and identified regulation predict physically active behavior in a way that is significantly mediated by intention, attitude, and perceived behavioral control. However, we also found a direct effect of intrinsic motivation on physical activity over time, which represents a partial mediation. Identified regulation directly affected the intention of becoming physically active, while the mediation through attitude and perceived behavioral control was also significant, thus representing another partial mediation. These findings are consistent with the findings of Teixeira, Carraça, Markland, Silva, and Ryan [50], who in their systematic review found that identified regulation predicted initial and short-term behavior, while intrinsic motivation rather predicted long-term exercise adherence. On the one hand, identified regulation relies on rather external but personally important values implemented to the self, which may explain a direct effect on intention rather than a long-term effect on physical activity. On the other hand, intrinsic motivation implies that being physically active is inherent by nature and, thus, in the long-term, directly related to the behavior itself. However, it should be noted that, while inherently perceiving the activity as being fun and interesting is important to sustain a long-term regular engagement in exercise, it requires engagement and effort to continue performing repetitive activities, which requires identification with the outcomes [51]. Furthermore, as expected, introjected regulation, external regulation, and amotivation were related to subjective norm, as externally controlled motivators are present in these constructs. Contrary to the theory of planned behavior, but in accordance with the findings of Hagger and colleagues [3], subjective norm was not significantly related to intention in our model. We assume that the internal motivators in autonomous motivation, attitudes, and perceived behavioral control explain substantially more variance in intention than the external drivers in introjected regulation and external regulation mediated by subjective norm, as also suggested by previous research [52]. In contrast to our assumption, beyond the expected significant path of controlled motivation types on subjective norm, intrinsic motivation and identified regulation were significant predictors. In fact, there is a disagreement in the scientific literature, as some scholars claim that the concept of subjective norms concerning social pressures is less likely to be aligned with autonomous motivation [52]. However, evidence for the above-mentioned path can be found in a recent meta-analysis [6]. The positive relation between autonomous motivation and subjective norm may be explained by the individuals’ recognition and respect of the desires of significant others and may be viewed as supportive and in accordance with self-endorsed values. Future studies should aim at investigating the particular relationship between autonomous motivation and subjective norm in more depth. As expected, amotivation negatively predicted attitude and positively predicted subjective norm; however, perceived behavioral control was also positively predicted. Even though amotivation represents the lack of any personal causal value of becoming physically active, this does not necessarily indicate that the students perceive to have no control over their behavioral actions. Thus, the students might perceive to have behavioral control over their actions but simply do not see any value in being active. In accordance with previous findings [53], we suggest that amotivation should be conceptualized in studies as a multidimensional construct, because different reasons may result in a lack of motivation (i.e., ability beliefs, effort beliefs, characteristics of the task, and value placed on the task).

On a more general level, we have seen that the different types of autonomous motivation are transferable from PE to LT, and vice versa. Consequently, this finding raises two questions. Firstly, how can PE teachers ideally motivate their students in a way that their autonomous motivation also persists in LT? Our results and previous findings suggest that the support of the psychological basic needs does increase autonomous motivation, as the fulfillment of basic needs is essential for ongoing psychological growth, integrity, and wellbeing [13]. Intervention programs—for instance, autonomy-supportive intervention programs for teachers [14]—have been successful in reducing controlling strategies in teaching. More specifically, five instructional behaviors have been found to be key elements: vitalizing rather than neglecting students’ inner motivational resources (e.g., using instructional strategies to spark interest in an intrinsic goal), relying on informational rather than on controlling language (e.g., saying ‘‘you may’’ rather than ‘‘you have to’’), providing instead of neglecting explanatory rationales, displaying patience rather than pressuring students, and acknowledging and accepting students’ complaints and expressions of negative affect. Furthermore, supporting their students’ autonomy allows the teacher to provide choice in order to impart freedom to determine their own behavior [54]. A sense of ownership and volition further enhances the feeling of being in control of one’s own actions [55]. Competence support refers to the ongoing provision of feelings of success in the students by the teacher, with, for example, guidance, optimal challenges for the individual student, and appropriate feedback. When teachers utilized these autonomy and competence supportive strategies, increased experiences of self-determination and intrinsic learning motivation of their students were found [48]. Moreover, the practical implications of relatedness support in class should be noted, as supportive relationships with teachers have been found to be key factors for students’ emotional engagement and academic achievement [56,57]. According to the students’ perception, the teacher’s communication (e.g., individualized and friendly), their in-class social support (e.g., promoting cooperation and teamwork), and behaviors associated with the teacher’s attentiveness (e.g., awareness and caring behaviors) were rated as highly relatedness-supportive in class [58]. Secondly, as motivational regulation in LT was related to the motivational regulation in PE, an additional path to foster an overall internal regulation might be the promotion of autonomous motivation and the support of basic needs at home. One potential consideration represents a home–school partnership, as, for instance, parents’ involvement, autonomy support, and structure contribute to higher autonomous motivation and engagement of their children [59]. Furthermore, another great opportunity for students is to participate in school-based after-school programs, which were linked to numerous positive development outcomes including higher values of perceived guidance, facilitated relations to peers, and the opportunity to pursue particular skills and competencies in an autonomous climate [60].

Ultimately, the trans-contextual model has proven to be a key concept to transfer students’ motivation in physical activity from the classroom to an out-of-school-context. Recent research has expanded this model by obtaining further evidence for the contextual transfer of motivation beyond the context of physical activity in school, as, for instance, autonomous motivation towards mathematics in school was found to be related to autonomous motivation towards math homework [61]. Furthermore, by means of our study, we have provided evidence for the perpetually present mechanisms of the trans-contextual model for the first time, by illustrating the links between motivational constructs of students to be persistent over time.

### Limitations and Suggestions for Future Research

One limitation of the present study is the analyses being restricted to two measurement points. Future research should further examine the theoretical assumptions on more occasions in order to provide additional support for the causal interactions of the proposed paths of the trans-contextual model. The CLPM contains a number of assumptions, which may make its results more difficult to interpret [22]. To represent the dynamic nature of life course theories, for example, three to four measurement occasions would provide insights into the model-implied developmental trajectory and would allow us to take into account the within-person stability properly. A further limitation of this study is the sole use of self-report measurements. Future research could implement objective measurements, as, for example, behavioral differences between subjectively and objectively measured physical activity have been found [62]. The same applies to the support of the basic psychological needs by the PE teacher, which may be analyzed through observations or teacher reports. Moreover, the questionnaire that we used to assess the students’ basic need support has not yet been empirically validated. Given the lack of available questionnaires capturing all three constructs of basic need support simultaneously, we adapted the questionnaire described in Standage and colleagues [30] and found good psychometric properties for our representative sample. However, in general, we would like to stress the need for upcoming studies to empirically validate a basic need support questionnaire incorporating autonomy support, competence support, and relatedness support. Furthermore, the two questionnaires (PLOC-R and BREQ-II) used in this study fell short of capturing “integrated regulation”. Since we aimed at assessing the students’ motivation in PE, the PLOC-R was the only available questionnaire at the time of our data collection. In order to differentiate accurately between motivation in the PE and LT context, we chose the BREQ-II for the LT context due to its similar overarching structure to the PLOC-R. Nevertheless, with regard to future studies, we would like to recommend the inclusion of “integrated regulation” in order to measure the full continuum of motivational regulation in the SDT. In addition, some differences with regard to motivation and basic psychological needs were found for the participants who dropped out. Thus, the dropout sample is representative of a specific population. However, our attrition rate was primarily due to students being absent on the second measurement occasion, for which motivational aspects were found to be a key factor in previous research [63]. A further limitation of the present study is that the conceptual questions were restrictively oriented toward the context of physical activity. However, the trans-contextual transfer of autonomous motivation might be present beyond the context of PE, which should be investigated accordingly in future studies.

## 5. Conclusions

In order to come back to the initial question about the trans-contextual modality of motivation, the results of the present study strongly support its transferability between PE and LT. We initially mentioned that one of the main goals in education is the transfer of the learned skills and competencies towards the everyday life of the students. Specifically in PE, one goal is to motivate the students to also remain or become physically active in an out-of-school context. The present study revealed that, in the long term, autonomy support, competence support, and relatedness support by the PE teacher positively influence autonomous motivation of students in PE. When considering the alarmingly low percentage of adolescents spending at least one hour of their LT in physical activity, which is related to numerous adverse health consequences [64,65], PE teaching represents an important domain to intervene. Nevertheless, beyond the assumption of the unidirectional impact of PE on LT, motivational regulation towards similar activities is trans-contextually interrelated. Thus, the promotion of motivational regulation styles in out-of-school contexts should not be ignored, as they are directly related to PE. Furthermore, we provided evidence that indeed the self-determination theory could be integrated into the theory of planned behavior; thus, autonomous motivation is related to physical activity behavior and intention, mediated by attitude and perceived behavioral control.

## Figures and Tables

**Table 1:** Means, standard deviations, and intercorrelations of the study variables.

	M	SD	1.	2.	3.	4.	5.	6.	7.	8.	9.	10.	11.	12.	13.	14.	15.	16.	17.	18.
1. Amotivation (PE)	2.31	1.35	‒																	
2. External Regulation (PE)	3.50	1.53	0.50 *	‒																
3. Introjected Regulation (PE)	3.21	1.58	0.27 *	0.50 *	‒															
4. Identified Regulation (PE)	5.13	1.41	−0.35 *	−0.04	0.24 *	‒														
5. Intrinsic Motivation (PE)	5.20	1.37	−0.45 *	−0.18 *	0.12 *	0.73 *	‒													
6. Amotivation (LT)	2.12	1.40	0.51 *	0.33 *	0.24 *	−0.12 *	−0.20 *	‒												
7. External Regulation (LT)	2.83	1.52	0.23 *	0.30 *	0.39 *	0.16 *	0.08 *	0.46 *	‒											
8. Introjected Regulation (LT)	3.64	1.62	0.01	0.16 *	0.36 *	0.31 *	0.23 *	0.12 *	0.42 *	‒										
9. Identified Regulation (LT)	5.06	1.29	−0.30 *	−0.11 *	0.10 *	0.50 *	0.49 *	−0.27 *	0.12 *	0.46 *	‒									
10. Intrinsic Motivation (LT)	5.37	1.40	−0.37 *	−0.19 *	0.02	0.50 *	0.58 *	−0.34 *	0.03	0.31 *	0.80 *	‒								
11. Autonomy support (PE)	5.00	1.10	−0.25 *	−0.03	0.07 *	0.50 *	0.48 *	−0.06 *	0.13 *	0.19 *	0.36 *	0.38 *	‒							
12. Relatedness support (PE)	5.37	1.12	−0.26 *	−0.06 *	0.04	0.45 *	0.48 *	−0.13 *	0.04	0.12 *	0.34 *	0.38 *	0.82 *	‒						
13. Competence support (PE)	5.38	1.20	−0.29 *	−0.06 *	0.04	0.50 *	0.50 *	−0.12 *	0.06 *	0.16 *	0.37 *	0.39 *	0.82 *	0.81 *	‒					
14. Attitudes	5.64	1.21	−0.31 *	−0.21 *	−0.06 *	0.33 *	0.38 *	−0.28 *	−0.06 *	0.12 *	0.45 *	0.52 *	0.25 *	0.24 *	0.26 *	‒				
15. Subjective norms	4.72	1.57	0.02	0.10 *	0.20 *	0.25 *	0.19 *	0.17 *	0.43 *	0.31 *	0.26 *	0.20 *	0.25 *	0.20 *	0.20 *	0.19 *	‒			
16. Perceived control	5.23	1.36	−0.17 *	−0.09 *	−0.01	0.31 *	0.31 *	−0.13 *	−0.002	0.18 *	0.43 *	0.47 *	0.32 *	0.30 *	0.30 *	0.39 *	0.22 *	‒		
17. Intentions	4.27	1.64	−0.21 *	−0.14 *	0.03	0.37 *	0.35 *	−0.18 *	0.08 *	0.26 *	0.58 *	0.56 *	0.28 *	0.26 *	0.28 *	0.48 *	0.23 *	0.54 *	‒	
18. Physical activity (LT)	3.17	1.90	−0.17 *	−0.14 *	−0.02	0.21 *	0.24 *	−0.17 *	−0.02	0.13 *	0.42 *	0.43 *	0.18 *	0.18 *	0.20 *	0.33 *	0.10 *	0.40 *	0.62 *	‒

Notes. * *p* < 0.05; PE = physical education context; LT = leisure-time context.

**Table 2:** Results of the generalized linear mixed model (GLMM) investigating the effects of autonomy support, relatedness support, and competence support in PE for intrinsic motivation and identified regulation when controlling for time, age, and sex.

	Intrinsic Motivation					Identified Regulation				
Fixed Effects	Estimate	SE	*β*	*t*	*p*	Estimate	SE	*β*	*t*	*p*
Intercept	1.76	0.22	0.00	7.94	<0.001	1.56	0.16	0.00	9.69	<0.001
Autonomy support	0.16	0.03	0.13	6.24	<0.001	0.19	0.03	0.14	6.91	<0.001
Relatedness support	0.19	0.03	0.16	6.63	<0.001	0.08	0.03	0.07	2.61	<0.01
Competence support	0.30	0.03	0.27	11.19	<0.001	0.40	0.03	0.35	13.70	<0.001
**Random Effects**	**Variance**	***SD***				**Variance**	***SD***			
Time	0.65	0.81				0.63	0.79			
Age	0.05	0.23				0.06	0.24			
Sex	0.06	0.25				0.01	0.11			
Observations	3362					3362				
Marginal R^2^	0.26					0.27				
Conditional R^2^	0.67					0.61				
